# In silico analysis of epitope-based vaccine candidate against tuberculosis using reverse vaccinology

**DOI:** 10.1038/s41598-020-80899-6

**Published:** 2021-01-13

**Authors:** Shaheen Bibi, Inayat Ullah, Bingdong Zhu, Muhammad Adnan, Romana Liaqat, Wei-Bao Kong, Shiquan Niu

**Affiliations:** 1 College of Life Science, Northwest Normal University, Lanzhou, 730070 Gansu China; 2 Lanzhou Center for Tuberculosis Research and Gansu Provincial Key Laboratory of Evidence Based Medicine and Clinical Translation, Lanzhou University, Lanzhou, 730000 China; 3 Institute of Pathogen Biology, School of Basic Medical Sciences, Lanzhou University, Lanzhou, 730000 Gansu China; 4 State Key Laboratory of Environmental Geochemistry, Institute of Geochemistry, Chinese Academy of Sciences, 99 Lincheng west Road, Guanshan Lake District, Guiyang, 550081 Guizhou China; 5 Department of Biochemistry, Faculty of Biological Sciences, Quaid-I-Azam University, Islamabad, Pakistan

**Keywords:** Computational models, Genome informatics, Protein design, Protein function predictions, Protein structure predictions, Software

## Abstract

Tuberculosis (TB) kills more individuals in the world than any other disease, and a threat made direr by the coverage of drug-resistant strains of *Mycobacterium tuberculosis* (Mtb). Bacillus Calmette–Guérin (BCG) is the single TB vaccine licensed for use in human beings and effectively protects infants and children against severe military and meningeal TB. We applied advanced computational techniques to develop a universal TB vaccine. In the current study, we select the very conserved, experimentally confirmed Mtb antigens, including Rv2608, Rv2684, Rv3804c (Ag85A), and Rv0125 (Mtb32A) to design a novel multi-epitope subunit vaccine. By using the Immune Epitopes Database (IEDB), we predicted different B-cell and T-cell epitopes. An adjuvant (Griselimycin) was also added to vaccine construct to improve its immunogenicity. Bioinformatics tools were used to predict, refined, and validate the 3D structure and then docked with toll-like-receptor (TLR-3) using different servers. The constructed vaccine was used for further processing based on allergenicity, antigenicity, solubility, different physiochemical properties, and molecular docking scores. The in silico immune simulation results showed significant response for immune cells. For successful expression of the vaccine in *E. coli*, in-silico cloning and codon optimization were performed. This research also sets out a good signal for the design of a peptide-based tuberculosis vaccine. In conclusion, our findings show that the known multi-epitope vaccine may activate humoral and cellular immune responses and maybe a possible tuberculosis vaccine candidate. Therefore, more experimental validations should be exposed to it.

## Introduction

*Mycobacterium tuberculosis* is a global pathogen that infects about 1.5 billion individuals and kills 1.3 million individuals per year^1^. About 10.0 million people with tuberculosis (TB) suffered in 2018, according to the World Health Organization (WHO) report. Besides, WHO survey data from around the world shows that 0.6 million cases of multidrug-resistant tuberculosis (MDR-TB) were reported, of which about 0.24 million deaths occurred^2–4^. The growth of MDR-TB strains results in the insufficient or irregular antibiotic intake and limited treatment of TB. Individuals with lower immunity are more resistant to infection and unable to respond effectively to the immune system. Therefore, more efforts are required to advance the production of new TB vaccines^5,6^. Most of the immune response to Mtb involves cell immunity, (CD4^+^ and CD8^+^ T cells)^7,8^. Both CD4^+^ and CD8^+^ T cells, once stimulated, secrete cytokines that cause an immune response. Cytotoxicity and lysis of infected cells are also mediated by the CD8^+^ cells. For *M. tuberculosis* elimination, effective T cell responses are essential. Complex pathogenesis, slow growth of Mtb, and dormant ability are critical tasks in developing successful therapies for TB^9^.

Since 1923, the Bacillus Calmette–Guérin (BCG) vaccine, an attenuated form of *Mycobacterium bovis*, has become the only established TB vaccine providing prophylaxis to be used globally. BCG efficacy ranges from 0 to 80% against adolescent pulmonary TB that protects against immunization for 10–20 years^10,11^. As a live-attenuated vaccine, BCG has a low safety because of the risks related with its use in immunocompromised individuals and the chance of returning the pathogen to its virulent state^12^. There are currently 16 TB vaccines in phase I, II, and III clinical trials, and some of them are a live attenuated form of Mtb^13^. Viral vector-based vaccines such as MVA85A and Crucell-Ad35/AERAS-402 will reduce the vaccine's efficacy before exposure to the vector^14^. Live prophylactic vaccines such as recombinant BCG VPM1002 and MTBVAC are also produced^7,8^, and can return to a pathogenic type. Inoculating patients with vaccines dependent on subunits decreases the risk of virulence reversal. The subunit vaccines (M72 and H4) consist of various Mtb antigens. However, lack of immunogenicity and are seldom capable of inducing immunity to long-term illness, requiring several vaccinations with adjuvants' addition.

M72/AS01 E that was primarily considered clinically safe in both healthy and TB-diagnosed adults. However, several volunteers encountered local reactions at injection spots during phase II, which prematurely terminated the research^15^. Given the success of peptide vaccines such as H4/IC31, peptide vaccines are regarded as stable and potentially potent TB vaccines. H4/IC31 had clinically safe in phase I studies that induced a robust immune response in healthy adults, and BCG vaccinated infants^16^. Peptide vaccines usually have higher safety profiles due to epitopes without reactogenic responses^17^ and low manufacturing charges^18^.

Different methods have been implemented to develop more effective novel TB vaccines, and among these methods, subunit vaccines have shown great promise^19^ i.e., the variety of epitopes in the Mtb protein antigens constituting the subunit vaccine. Besides computational studies, some studies had endeavored to evaluate the human T cell immune responses to multiple Mtb subunit vaccines empirically^20,21^ and using data from clinical trials, respectively^22–24^. Rodo et al. recommended that the identification of vaccines with distinctive immune response features may increase the probabilities of finding a safe vaccine^21^ and provided valuable information on the potential production of the Mtb vaccine.

This study aimed to develop a TB epitope ensemble vaccine for new access in reverse vaccinology. The method has recently started to be demonstrated by the discovery of the epitope ensemble vaccine against SARS-CoV-2^25–27^, Ebola virus^28^, malaria^29^ *Acinetobacter baumannii*^30^, and *Staphylococcus aureus*^31^. Similarly, various immuno-informatics methods were used to develop a probable vaccine coding in the Mtb H37Rv genome for multiple B and T cell epitopes. They could potentially activate both humoral and cellular immunity^32^. Our finding proposed that the selected epitopes from the four Mtb antigens [Rv2608, Rv2684, Rv3804c (Ag85A), and Rv0125 (Mtb32A)] could be used effectively as potential candidates for vaccines and will be applied for future experimental research to eradicate TB. We selected Mtb epitopes with demonstrated immunogenicity combined to form an effective, widely available epitope ensemble vaccine, establishing a universal vaccine.

## Materials and methods

### Selection of Mtb strain, antigens and retrieval of protein sequences

There are 7 phylogenetic branches of Mtb, with lineages 2, 3, and 4 being responsible for the majority of worldwide spread. Most preclinical work has used lineage 4 derived vaccines due to the common lab strain H37Rv being of lineage 4. However, there was little direct evidence to support this selection^33^. The Immune Epitope Database and Analysis Resource (IEDB; http://www.iedb.org/) was used to collect *M. tuberculosis*-specific epitopes. We selected the antigens, based on their antigenicity, immunogenicity, conservancy, MHC binding affinity and IFN-γ stimulation. Antigens have chosen for this study included; Rv2608 (Accession No.: P9WHZ5), Rv2684 (Accession No.: P9WPD9), Rv3804c (Accession No.: P9WQP3) and Rv0125 (Accession No.: O07175). The Mtb H37Rv protein amino acid sequences were obtained from the mycobrowser database (https://mycobrowser.epfl.ch/). For all species of Mycobacteria, Mycobrowser is the complete genomic and proteomic database^34^ including *M. tuberculosis*,* M. leprae*,* M. marinum*, and *M. smegmatis*^35^. Further analysis was done with the assistance of these protein sequences.

### Prediction of cytotoxic T lymphocytes epitope

For the production of the subunit vaccine, cytotoxic T lymphocyte (CTL) epitope prediction is very important. The amino acid sequence was analyzed using NetCTL 1.2 server (https://www.cbs.dtu.dk/services/NetCTL/) for the prediction of the CTL epitopes^36^. Prediction of the epitopes depends on three main qualities firstly of MHC-I binding affinity, second, proteasomal C terminal cleavage performed using artificial neural networks (ANN), and third, TAP (Transporter Associated with Antigen Processing) transport efficiency which was predicted using weight matrix. For the prediction of the CTL epitopes the thresholds for different parameters like TAP transport efficiency, proteasomal C-terminal cleavage, and epitope identification was set 0.05, 0.15 and 0.75, respectively. The predicted epitopes were categorized according to the combined score. Although the server allows for CTL epitopes predictions limited to 12 MHC class I supertypes, only the A1 supertype was used for this study^37^.

### Prediction of Helper T lymphocytes epitope

The IEDB MHC II server (http://tools.iedb.org/mhcii/) was used for the prediction of Helper T lymphocytes (HTL) epitopes^38^. The species/locus was chosen as Human/HLA-DR, and a 7-allele human leukocyte antigen (HLA) reference set was selected for the HTL epitopes prediction. Further, 15-mer length of the epitopes were retrieved and classified according to the percentile value. The percentile rank is given after comparing the peptides score with 5 million 15-mer from the SWISSPROT database, compounds with the least percentile rank show a high affinity of MHC-II.

### Prediction of interferon-gamma inducing epitopes

The chosen HTL epitopes were submitted to investigate whether they can induce interferon-gamma (IFN-γ) immune response by using the (15-mer) IFN-gamma epitope server (http://crdd.osdd.net/raghava/ifnepitope/scan.php). The server constructs overlapping sequences from which the IFN-γ epitopes are predicted, and prediction based on Support Vector Machine (SVM) and model was predicted by selecting IFN-γ versus non-IFN-γ^39^. Finally, for the in silico vaccine construction, the epitopes with positive results for the IFN-γ response were selected.

### Prediction of linear B-cell epitopes

B-cell epitopes are essential for stimulating a humoral immune response, which activates B lymphocytes for antibody production and plays a dynamic role in vaccine designing. The antigens were exposed to linear B-cell epitope prediction using ABCpred servers (http://www.imtech.res.in/raghava/abcpred/)^40^. For the identification of the epitopes, the window length was chosen to be 16-mer, based on recurrent neural network with a 0.51 threshold value, keeping overlapping filter on. Top predicted epitopes having score more than 0.9 was only chosen for the construction of the candidate vaccine.

### Prediction of antigenicity of proteins sequences

It is a significant feature of vaccine developing that designated vaccine candidates must have antigenic property. ANTIGENpro and VaxiJen v2.0 both were used to measure the antigenicity of the vaccine candidates. ANTIGENpro (http://scratch.Proteomics.ics.uci.edu/), which uses micro-array data to calculate protein antigenicity. The server's accuracy with the combined dataset was calculated to be 76% based on cross-validation experiments^41^. While the antigenic evaluation of the selected genes was performed via a freely accessible online VaxiJen 2.0 server (http://www.ddg-pharmfac.net/Vaxijen/VaxiJen/VaxiJen.html) with a threshold value of ≥ 0.4^42^, only probable antigen epitopes were selected for the vaccine construction. VaxiJen 2.0 server is based on auto and cross-covariance (ACC) transformation of protein sequences into uniform vectors of major amino acid properties was used to evaluate the antigenicity of the vaccine. The VaxiJen algorithm is mainly based on the method of sequence alignment and analyzes protein physiochemical properties to identify them as antigenic^43^.

### Prediction of allergenicity and toxicity of proteins sequences

Allergen identification is a vital factor in the development of the vaccine. AllerTOP v.2.0 and AllergenFP servers measured the allergenic properties of the proteins. AllerTOP v2.0 an online server (http://www.ddg-pharmfac.net/AllerTOP) develops the k nearest neighbors (kNN), auto- and cross-covariance (ACC) transformation, and amino acid E-descriptors machine learning techniques for the classification of allergens by exploring the physiochemical properties of proteins. The accuracy of this approach was stated as 85.3% at fivefold cross-validation^44^. On the other hand, AllergenFP (http://ddg-pharmfac.net/AllergenFP/) is an alignment-free, descriptor-based fingerprint method for the detection of allergens and non-allergens. This method is based on a four-step algorithm. Initially, the protein sequences are defined in terms of their properties, including size, hydrophobicity, relative abundance, α helix and β-strand forming propensities. The generated strings that vary in length are transformed into vectors of equal size by ACC. The vectors were translated into binary fingerprints and measured according to the Tanimoto coefficient. The method was applied to known non-allergens and allergens and correctly recognized 88% of them with a Matthews correlation coefficient of 0.759^45^. Moreover, it is also a comprehensive and accurate method for the allergen prediction and also used by the researchers to predict allergens in the process of vaccine construction^46^. For further analysis, the protein sequences which are non-allergenic in the properties were selected.

And finally, all the epitopes were checked for toxicity using the ToxinPred server (https://webs.iiitd.edu.in/raghava/toxinpred/multi_submit.php)^47^, and non-toxic epitopes were chosen. The overall construct of the vaccine has also been verified for these characteristics.

### Construction of multi-epitope vaccine candidate sequence

The highly antigenic, immunogenic, non-toxic, and non-allergenic epitopes were selected for the final vaccine construct. Selected CTLs, HTLs epitopes, and B-cell epitopes predicted by using NetCTL 1.2, IEDB MHC II server and ABCpred server respectively, were used to construct multi-epitope vaccine sequence. The linear B-cell and HTL epitopes were linked with GPGPG linker and CTL epitopes by AAY linker. In addition, a griselimycin (APD ID: AP02688)^48^ was selected as an adjuvant to increase the immunogenicity of the vaccine, and linked via EAAAK linker. The sequence of the griselimycin was retrieved from the Antimicrobial Peptide Database (http://aps.unmc.edu/AP/main.php).

### Physiochemical properties and solubility prediction

Expasy Protparam (https://web.expasy.org/protparam/) was used to predict various physicochemical properties like theoretical isoelectric point (pI), amino acid composition, in vitro and in vivo half-life, instability and aliphatic index, molecular weight (MW), and grand average of hydropathicity (GRAVY) of the vaccine constructs^49^. The multi-epitope vaccine solubility was predicted using the Protein–Sol server (http://protein-sol.manchester.ac.uk). The scaled solubility value (QuerySol) is the predicted solubility. The population average for the experimental dataset (PopAvrSol) is 0.45, and thus any scaled solubility value greater than 0.45 is predicted to have a higher solubility than the average soluble *E. coli* protein from the experimental solubility dataset^50^. The protein with a lower scaled solubility value is predicted to be less soluble.

### Secondary structure prediction

The secondary structures of the vaccine constructs were generated using the online tool PSIPRED and RaptorX Property servers. PRISPRED (http://bioinf.cs.ucl.ac.uk/psipred/), is an online server secondary structure generating tool that also predicts the transmembrane topology, transmembrane helix, fold and domain recognition etc efficiently^51^. The RaptorX Property (http://raptorx.uchicago.edu/StructurePropertyPred/predict/) was additional used to calculate the secondary structure characteristics of the vaccine. The server uses an evolving machine learning model called Deep Convolutional Neural Fields (Deep CNF) to continuously calculate secondary structure (SS), disorder regions (DISO), and solvent accessibility (ACC)^52^.

### Tertiary structure prediction

The tertiary or three-dimensional (3D) model of the multi-epitope vaccine was prepared using the homology modeling tool I-TASSER (Iterative Threading ASSEmbly Refinement) server (https://zhanglab.ccmb.med.Umich.Edu/I-TASSER/). The I-TASSER server is an integrated platform for computerized protein structure and function prediction based on the sequence-to-structure-to-function paradigm and identifies similar structure patterns from the Protein Data Bank (PDB)^53^. I-TASSER initial produces 3D atomic models from several threading alignments and iterative structural assembly simulations starting from an amino acid sequence. A template modeling (TM)-value > 0.5 shows a model of accurate topology, and a TM-value < 0.17 indicates a random similarity. These cutoff value does not depend on the length of the protein^54^. In the previous five community wide CASP (Critical Assessment of techniques for Structure Prediction) experiments, I-TASSER was ranked finest server for protein 3D structure prediction^55^.

### Refinement of the tertiary structure

GalaxyRefine web server (http://galaxy.seoklab.org/cgi-bin/submit.cgi?type=REFINE) has refined the 3D model obtained for the multi-epitope vaccine peptide. The GalaxyRefine server is based on a refinement approach that was effectively verified in CASP10 based refinement experiments. and achieves the repacking and molecular dynamics simulation to relax the structure. This method can improve the quality of both global and local structures when used to improve the models produced by state of the art protein structure prediction servers^56^.

### Validation of tertiary structure

Tertiary structure validation is a severe stage of the model construction method because it identifies possible mistakes in 3D models predicted^57^. ProSA-web server (https://prosa.services.came.sbg.ac.at/prosa.php) was initially used for protein 3D structure validation, which estimates a total quality score exact input structure, which is shown in the form of Z score. If the Z scores are outside the range of the properties for native proteins, it specifies that the structure likely contains errors^58^. To investigate non-bonded atom–atom interactions associated with the ERRAT web-server (http://services.mbi.ucla.edu/ERRAT/) was also used to predict high-resolution crystallography structures. A Ramachandran plot was retrieved via RAMPAGE web-server (http://mordred.bioc.cam.ac.uk/~rapper/rampage.php) and describe the quality of the modeled structure by displaying the percentage of residues in disallowed and allowed regions^59^.

### Prediction of discontinuous B-cell epitopes

More than 90% of B cell epitopes were determined to be discontinuous. ElliPro, an online server (http://tools.iedb.org/ellipro/), has been used to predict the validated 3D structure of discontinuous (conformational) B-cell epitopes. ElliPro implements three algorithms based on their protrusion index (PI) values to estimate the protein shape as an ellipsoid, measure the residue PI, and adjacent cluster residues. ElliPro offers a score for each output epitope termed as an average PI value over each epitope’s residue. The ellipsoid with a PI value of 0.9 contains (90%) protein residues included while the (10%) residues are outside ellipsoids. The PI value for each epitope residue was determined based on the center of residue mass residing outside the largest ellipsoid possible. Compared to other structure based approaches used to predict epitopes, ElliPro achieved the top and provided an AUC value of (0.732) as the best calculation for any protein.

### Molecular docking of the final vaccine with immune receptor

It is based on the interface between an antigenic molecule and a particular immune receptor to produce an effective immune response. TLR3 (PDB ID: 1ZIW) was downloaded from Protein Databank (PDB) (https://www.rcsb.org). Online servers ClusPro 2.0 (https://cluspro.bu.edu/login.php), HADDOCK server (https://haddock.science.uu.nl/), PatchDock server (https://bioinfo3d.cs.tau.ac.il/PatchDock/php.php), and FireDock server (http://bioinfo3d.cs.tau.ac.il/FireDock/php.php) were used for molecular docking and docking refinement, respectively^60^. Again, the docking was performed for the third time using the HawkDock server (http://cadd.zju.edu.cn/hawkdock/), and subsequently, the Molecular Mechanics/Generalized Born Surface Area (MM-GBSA) score was also measured using the same server that predicts the result in the affinity score and the lowest prediction score is considered the better score^61^.

### Molecular dynamics simulation

The molecular dynamics simulation study was conducted for the vaccine construct that showed the best molecular docking study results. The iMODS web-server (http://imods.Chaconlab.org/) was used for the molecular dynamics simulation study, a fast, free-accessible and molecular dynamics simulation server for defining and calculating the protein flexibility^62^.

### Codon optimization and in silico cloning

A codon optimization approach was used to improve recombinant protein expression. Codon optimization is essential because the genetic code's degeneracy permits most of the amino acids to be encoded by multiple codons. Java Codon Adaptation Tool (JCat) server (http://www.prodoric.de/JCat) was used in the codon system of *E. coli* (strain K12) to obtain the codon adaptation index (CAI) values and GC contents to determine the levels of protein expression. The best CAI value is 1.0, while > 0.8 is regard a good score, and the GC content range from 30 to 70%. There are unfavorable effects on translation and transcriptional efficiencies beyond this range^63^. The multi-epitope vaccine's optimized gene sequence was cloned in *E. coli* plasmid vector pET-30a (+), NdeI and HindIII restriction sites were added to the N and C-terminals of the sequence, respectively. Finally, the optimized sequence of the final vaccine construct (with restriction sites) was inserted into the plasmid vector pET-30a (+) using the SnapGene software (https://www.snapgene.com/free-trial/) to confirm the expression of the vaccine.

### Immune simulation

In silico method C-ImmSim, online simulation server (http://150.146.2.1/C-IMMSIM/index.php) reported the vaccine constructs immune response profile. C-ImmSim defines a mammalian immune system's both humoral and cellular response to the vaccine construct^64^.Three injections of the target product profile of the prophylactic tuberculosis vaccine were administered at different intervals of 4 weeks. All simulation parameters were established at default with time periods set at 1, 84, and 168. The volume of simulation and the steps of the simulation were set at 50 and 1000, respectively. (random seed = 12345 with vaccine injection not containing LPS.

## Results

### Protein sequences and PDB structures

The amino acid sequence of *Mycobacterium tuberculosis* H37Rv was saved from the mycobrowser database as a FASTA format. Hence, then the functional sequences for the four proteins were subjected to linear B-cell and T-cell epitope prediction and develop a novel subunit vaccine against tuberculosis.

### Prediction of cytotoxic T lymphocytes epitope

For the four nominated proteins, 34 CTL (9-mer) epitopes were predicted using the NetCTL 1.2 web-server fixed at the threshold value for epitope documentation. Out of all these predicted CTL epitopes, only ten epitopes were chosen to construct the vaccine based on their high scores binding affinity towards MHC-I, antigenicity, non-allergenicity, and non-toxicity, as illustrated in (Table 1).

**Table 1:** List of the selected CTL epitopes which have fulfilled all the criteria for antigenicity, non-allergenicity, non-toxicity and could bind efficiently to MHC-I A1-supertype alleles.

Serial. No.	Peptide sequence	MHC binding affinity	Rescale binding affinity	C-terminal cleavage affinity	Transport efficiency	Prediction score	Protein
1	TIATFEMRY	0.3660	1.5541	0.9655	3.2030	1.8591	Rv2608
2	SSPDVLTTY	0.3629	1.5410	0.9785	3.0500	1.8402	
3	PTVDYAFQY	0.3519	1.4942	0.9752	2.4490	1.7629	
4	STDTPWWAL	0.4481	1.9027	0.9738	0.6650	2.0820	Rv2684
5	VSIALAAIY	0.2959	1.2563	0.1246	3.2500	1.4375	
6	SVEWDTLLF	0.2028	0.8610	0.8627	2.6360	1.1222	
7	DSGTHSWEY	0.6403	2.7186	0.9393	2.3410	2.9766	Rv3804c
8	SSALTLAIY	0.4867	2.0664	0.8423	3.1890	2.3522	
9	ATDINAFSV	0.3986	1.6923	0.9423	0.2310	1.8452	Rv0125
10	FADFPALPL	0.1357	0.5762	0.8727	0.8680	0.7505	

### Prediction of Helper T lymphocytes epitope

High-binding MHC-II epitopes for human alleles HLA-DR, predicted with the IEDB MHC-II web server were defined as HTL epitopes. A total of four HTL epitopes were nominated for the final vaccine on the basis of binding affinity, antigenicity, non-allergenicity, and non-toxicity, as illustrated in (Table 2). Human alleles and position of predicted epitopes are HLA-DRB3*01:01(45–59), HLA-DRB1*03:01(113–127), HLA-DRB3*02:02 (270–284) and HLA-DRB3*02:02 (406–420).

**Table 2:** List of the final selected HTL epitopes which fulfilled all the criteria for antigenicity, non-allergenicity, non-toxicity and could also induce the IFN-γ immune response.

Serial no.	protein	Allele	Start	End	Peptide sequence	Percentile score	Method	Result	IFN-γ score
1	Rv2608	HLA-DRB3*02:02	406	420	PQLGFTLSGATPADA	1.5	SVM	Positive	1
2	Rv2684	HLA-DRB3*01:01	45	59	IFYSHDTGIDWDVIF	0.84	SVM	Positive	1
3	Rv3804c	HLA-DRB3*02:02	270	284	KFLEGFVRTSNIKFQ	2.6	SVM	Positive	1
4	Rv0125	HLA-DRB1*03:01	113	127	TYGVDVVGYDRTQDV	3.4	SVM	Positive	11

### Prediction of interferon-gamma inducing epitopes

The IFN-gamma plays a significant role in intracellular pathogen evasion and majorly acts as cytokines for cytotoxic T lymphocytes and natural killer cells. The IFN-γ inducing epitopes predicted by the IFN-γ epitope server, using the Support Vector Machine (SVM) method. Four HTL epitopes with IFN-γ positive scores were chosen for vaccine construction.

### Prediction of linear B-cell epitopes

ABCpred server was used to predict the B cell epitopes. All predicted B-cell epitopes (16-mer) with a cut-off binding score > 0.9, high antigenic, non-allergenic and non-toxic, a total of four B-cell epitopes finally chosen for vaccine construction, are listed in Table 3.

**Table 3:** Predicted linear B cell epitopes, a binding score greater than 0.9, are only selected for the final vaccine construct.

Serial no.	Protein	Peptide sequence	Start position	Predicted score
1	Rv2608	GITGNGQIGFGKPANP	241	0.95
2	Rv2684	SRAGLTFNDFMLHLTP	164	0.94
3	Rv3804c	YSDWYQPACGKAGCQT	122	0.93
4	Rv0125	PALPLDPSAMVAQVGP	46	0.93

### Construction of multi-epitope subunit vaccine

**Figure 1:**
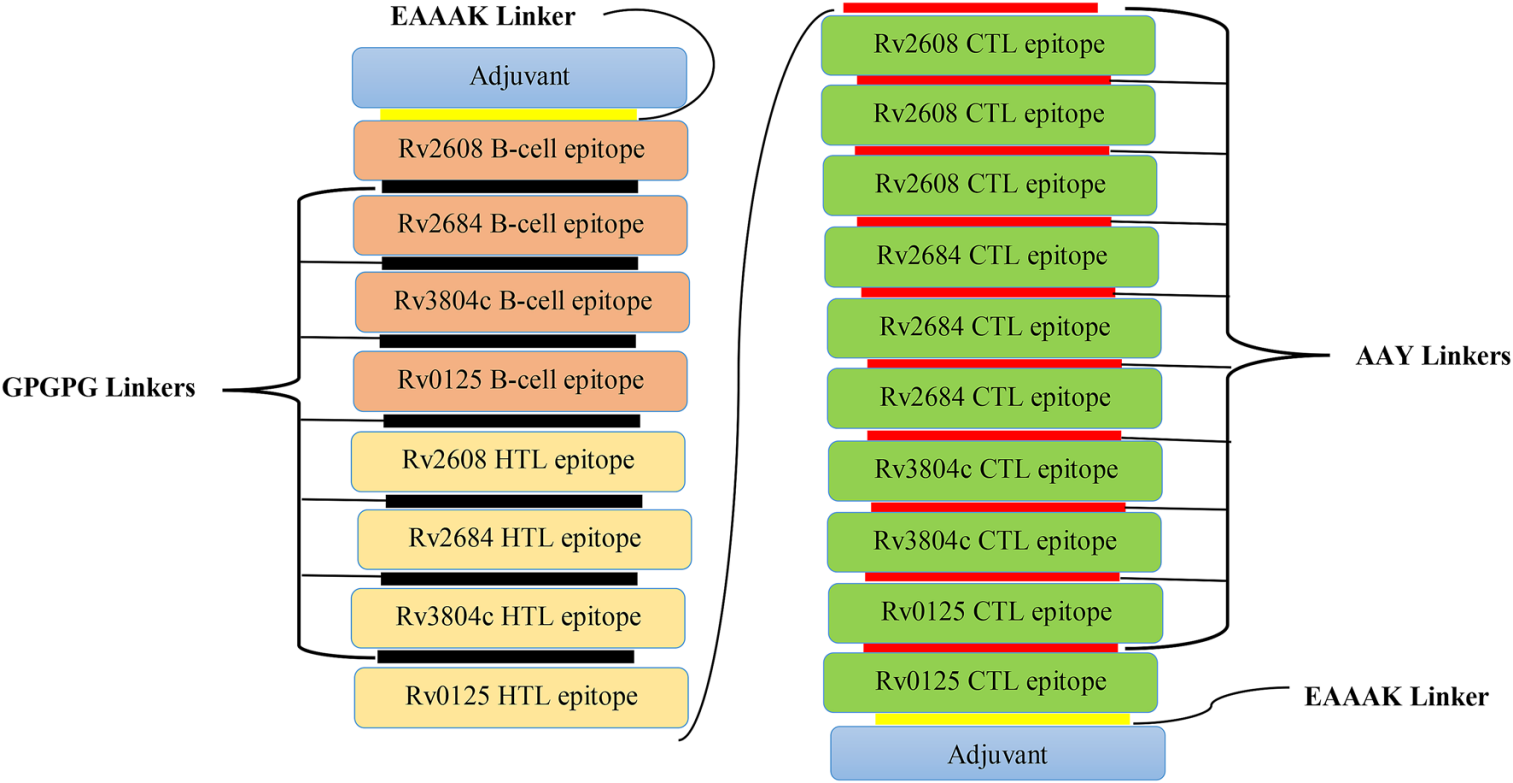
Schematic presentation of the final multi-epitope vaccine peptide. The 309-amino acid long peptide sequence containing adjuvant (blue) at both N and C terminal was linked with the multi-epitope sequence through an EAAAK linker (yellow). B cell epitopes and HTL epitopes are linked using GPGPG linkers (black) while the CTL epitopes are linked with AAY linkers (red).

Four B cell epitopes, four HTL epitopes, and ten CTL epitopes were nominated to design a novel vaccine, fulfilling the criteria of binding affinity, antigenicity, non-toxicity, and non-allergenicity. In addition to these epitopes, to improve immunogenicity, an adjuvant griselimycin with APD ID: AP02688 also applied to both the N and C terminals of the vaccine. EAAAK linkers link adjuvant to the epitopes, GPGPG linkers were used to link B-cell and HTL epitopes and AAY linkers to link CTL epitopes. The constructed vaccine sequence was again tested for antigenicity, non-allergenicity, non-toxicity, solubility and fulfilling all the criteria. The schematic presentation of the final multi-epitope vaccine peptide of the current study is defined in Fig. 1.

### Prediction of antigenicity and allergenicity of the antigens and vaccine candidate

The four proteins were investigated for the antigenicity by ANTIGENpro and VaxiJen v2.0 web tool, and it was found that all the four chosen proteins could be good antigens. In the VaxiJen v2.0 tool, the default threshold of 0.4 was chosen as the antigenicity criterion. The antigen Rv2608, Rv2684, Rv3804c and Rv0125 showed an antigenicity score of 0.54, 0.46, 0.52 and 0.81 respectively. The antigenicity of the vaccine was predicted by the VaxiJen 2.0 server to be 0.81 and 0.66 with ANTIGENpro. The results specify that the constructed vaccine is highly antigenic. The vaccine sequence on both the AllerTOP v.2 and AllergenFP servers is both estimated to be non-allergenic.

### Prediction of physiochemical properties and solubility

**Figure 2:**
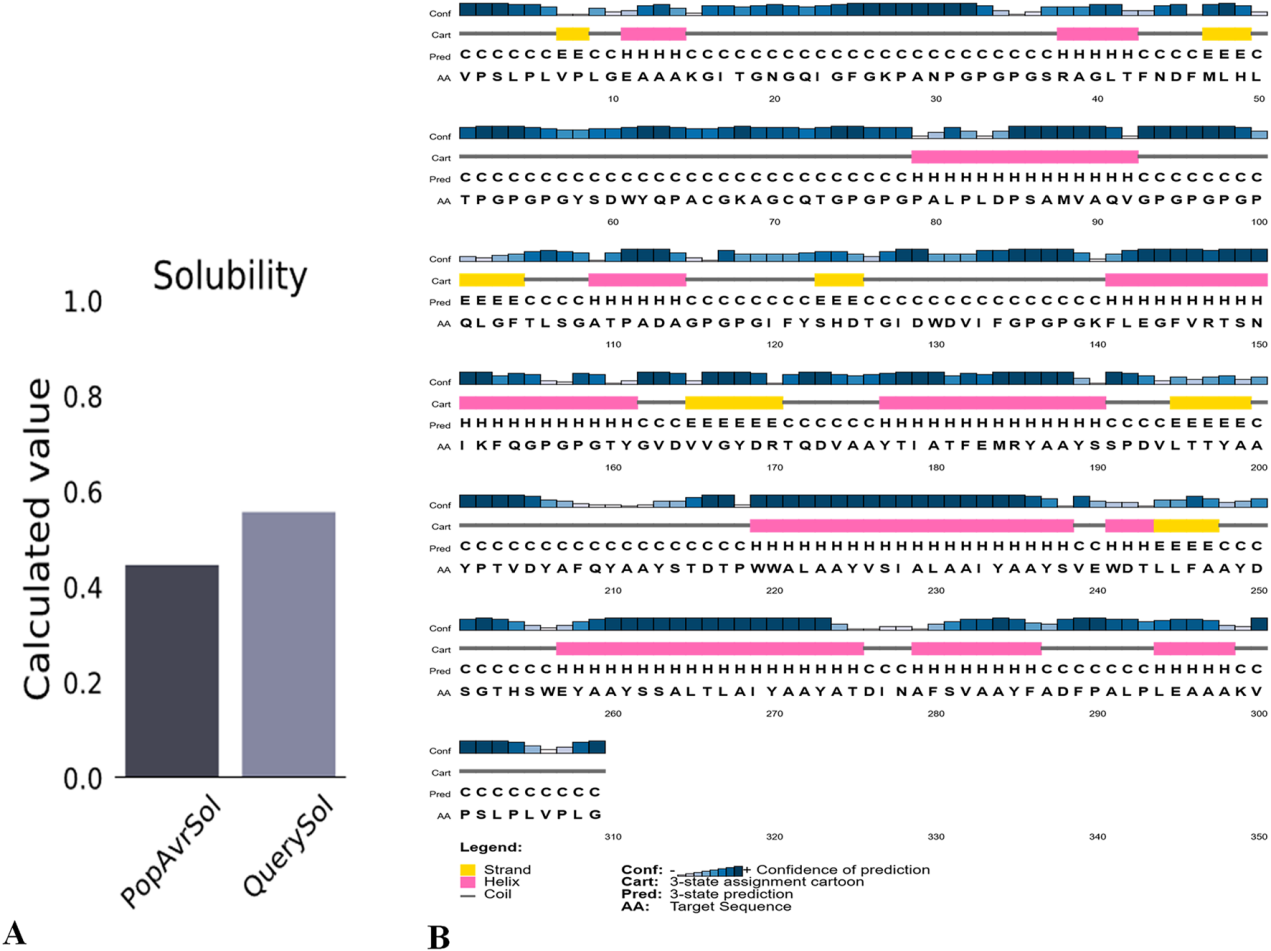
Solubility analysis and secondary structure predictions of vaccine construct. (**A**). Solubility analysis of vaccine constructs using ProtSol with a score of 0.557 upon expression. (**B**). Secondary structure prediction of vaccine constructs using the PSIPRED server having (20.0%) alpha-helices, (21.0%) beta-strands, and (58.0%) coils.

The final protein's molecular weight was estimated to be 31.9 kDa with a theoretical isoelectric point (pI) score of 4.28. It was estimated that the half-life was 100 h in mammalian reticulocytes in vitro and more than 20 h in yeast and about 10 h in *E. coli* in vivo. The instability index (II) value was 25.51, suggesting the protein is extremely stable. (II of > 40: instability). The high aliphatic index score of 74.40 indicates thermos ability^65^. The Grand average of hydropathicity was found 0.116, which shows a hydrophilic nature of the vaccine constructs. The solubility score of protein was 0.557, which indicates the protein is highly soluble upon expression (Fig. 2A).

### Prediction of secondary structure

The overall vaccine sequence was estimated to have 20% α-helix, 21% β-strand, and 58% coil (Fig. 2B). Furthermore, 38% of amino-acid residues were expected to be exposed, 22% medium exposed, and 38% buried in support of solvent accessibility.

### Tertiary structure modeling

**Figure 3:**
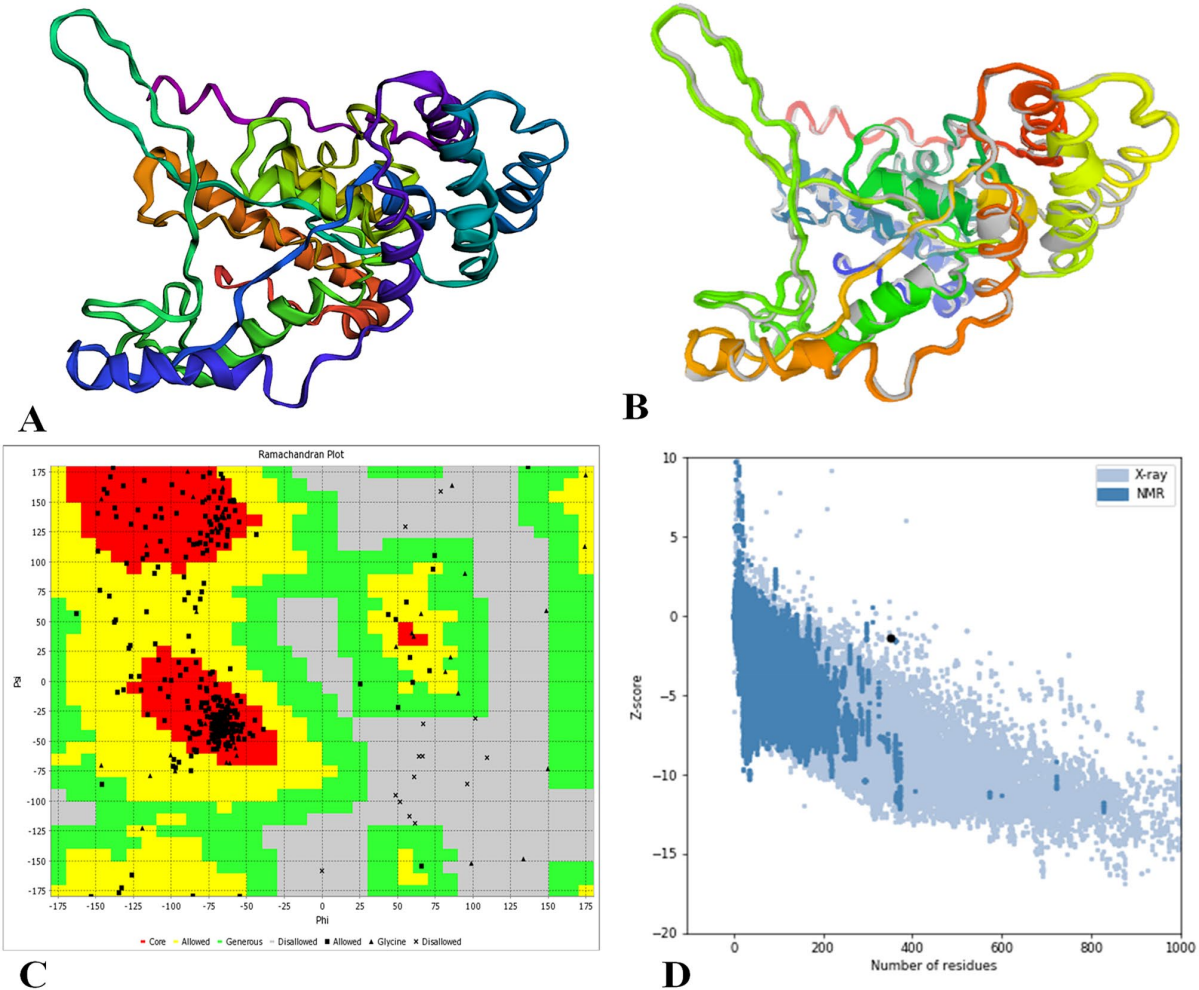
Protein 3D modeling, refinement, and validation. (**A**) The 3D model of a multi-epitope vaccine was obtained on the I-TASSER server following homology modeling. (**B**) Refinement: superimposition by the GalaxyRefine server of a refined 3D structure (colored) on a ‘crude model’ (gray). (**C**) Validation: Ramachandran plot analysis showing 85.9% in favored, 8.9% in allowed, and 5.2% in disallowed regions of protein residues and (**D**) ProSA-web, with a Z score of − 1.39.

Five tertiary 3D structures of the designed vaccine were predicted by the I-TASSER web-server based on ten threading templates, with Z score values (1.10–2.78) and confidence score (C-score) values (− 3.96 to − 1.31). Usually, the C score series is from − 5 to 2, with high scores representing high sureness. The best structure with the C value − 2.01 from the modelling chosen for additional analysis. (Fig. 3A). This structure had a probable TM-score of 0.47 ± 0.15, with an expected root-mean-square deviation (RMSD) score of 11.2 ± 4.6 Å. The TM-value has been recommended as a calculating scale for the structural resemblance among the structures. The TM-value was suggested to address the issue of RMSD, that is delicate to native mistake.

### Tertiary structure refinement

The GalaxyRefine web-server was used to enhance the consistency of the modeled protein. The loop refinement and energy minimization were carried out to obtain the high quality of the predicted structure. The refinement of the initial “crude” vaccine model on the GalaxyRefine web-server produced five model structures. Based on structure quality for all developed structures, model 4 was the most significant based on several factors, i.e., GDT-HA (0.9079), RMSD (0.518), and MolProbity (2.510). The clash-value was 22.4, the low rotamers-value was 0.4, and Rama favored value was 84.50. This model was selected for additional study (Fig. 3B).

### Tertiary structure validation

The refined structure was exposed to the Ramachandran plot analysis using the RAMPAGE web-server. The VADAR web tool plot exposed 85.9% of residues in favored regions, and 8.9% is allowed regions 5.2% of residues in the outlier region (Fig. 3C). Both ProSA-web and ERRAT verified the quality and potential errors in the crude 3D model. The chosen model after refinement had an overall quality factor of 87.9% with ERRAT. The Z score for the input vaccine was estimated to be − 1.39 by the ProSA-web-server (Fig. 3D). The overall results from RAMPAGE, ERRAT, VADAR web tool, and ProSa-web have validated the 3D modeled protein's outstanding quality.

### Prediction of conformational B-cell epitopes

**Figure 4:**
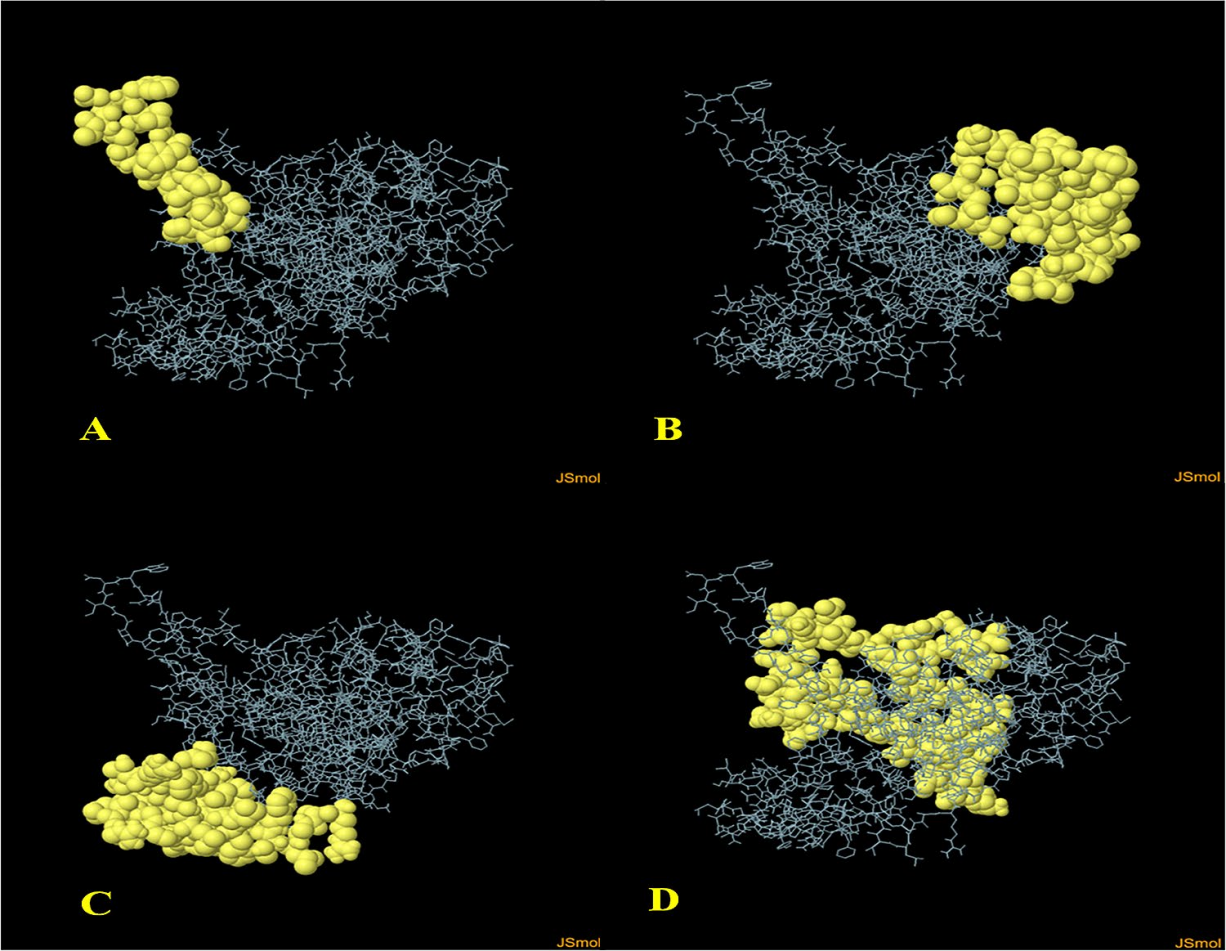
Three-dimensional representation of conformational or discontinuous B cell epitopes of the designed multi-epitope based vaccine. (**A–D**) A yellow surface represents the conformational or discontinuous B cell epitopes, and the bulk of the polyprotein is represented in grey sticks.

**Figure 5:**
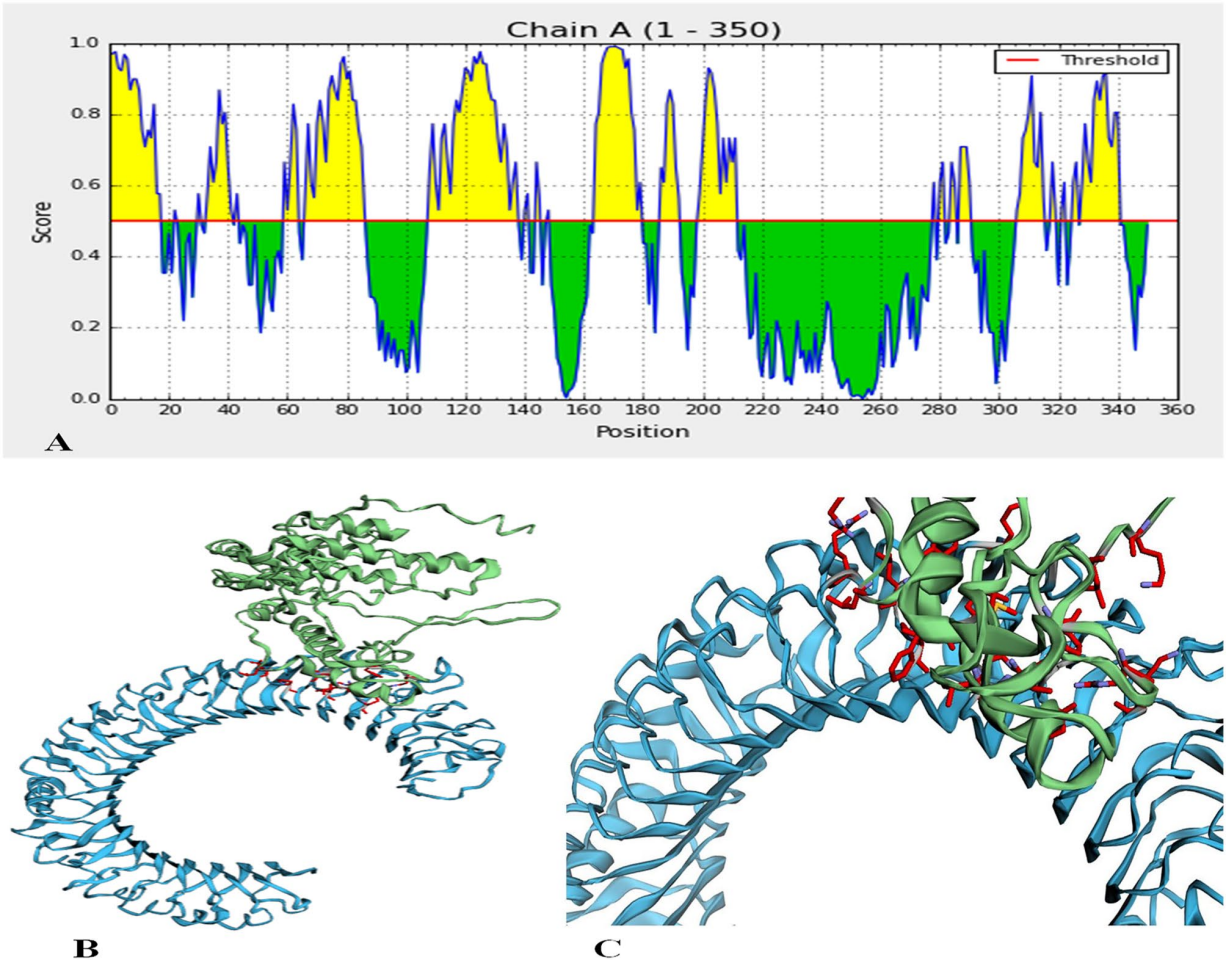
Discontinuous B cell epitopes and the interaction between the ligand protein, (multi-epitope subunit vaccine) and receptor protein, (TLR-3). (**A**)The individual score of discontinuous B cell epitopes was predicted in the multi-epitope subunit vaccine. (**B**,**C**) The ligand protein is indicated by green color and the receptor protein is indicated by blue color.

One hundred ninety-two residues were estimated to be situated in four discontinuous B-cell epitopes, with values from 0.69 to 0.785. The conformation epitopes ranged in size from 20 to 65 residues. For discontinuous peptides that predict using Ellipro, the score value of 0.69 or more was selected (Fig. 4A–D) and (Table 4). And various discontinuous epitope residues were predicted from vaccine sequence length 161–180 (20 epitope residues), between 33–45, 107–137, 140–141 and 144–149 (52 epitope residues), between 59–64, 66–88, 183, 185–193, 196–211 (55 epitope residues), between 1–18, 277–278, 280–281, 284–285, 287–293, 295, 304–319, 321–322, 325–326, 328–340 and 342 (65 epitope residues) were predicted. The individual score of each of the discontinuous epitopes has been shown in (Fig. 5A).

**Table 4:** ElliPro predicted the conformational B cell epitopes residues of the designed multi-epitope based vaccine.

No.	Residues	Number of residues	Score
1	A:I161, A:F162, A:Y163, A:S164, A:H165, A:D166, A:T167, A:G168, A:I169, A:D170, A:W171, A:D172, A:V173, A:I174, A:F175, A:G176, A:P177, A:G178, A:P179, A:G180	20	0.785
2	A:P33, A:G34, A:P35, A:G36, A:F37, A:A38, A:S39, A:V40, A:T41, A:T42, A:G43, A:L44, A:A45, A:S107, A:A108, A:M109, A:V110, A:A111, A:Q112, A:V113, A:G114, A:P115, A:G116, A:P117, A:G118, A:P119, A:G120, A:P121, A:Q122, A:L123, A:G124, A:F125, A:T126, A:L127, A:S128, A:G129, A:A130, A:T131, A:P132, A:A133, A:D134, A:A135, A:G136, A:P137, A:G140, A:T141, A:F144, A:L145, A:E146, A:T147, A:P148, A:S149	52	0.692
3	A:R59, A:A60, A:G61, A:L62, A:T63, A:F64, A:D66, A:F67, A:M68, A:L69, A:H70, A:L71, A:T72, A:P73, A:G74, A:P75, A:G76, A:P77, A:G78, A:R79, A:A80, A:L81, A:G82, A:A83, A:T84, A:P85, A:N86, A:T87, A:G88, A:L183, A:G185, A:F186, A:V187, A:R188, A:T189, A:S190, A:N191, A:I192, A:K193, A:G196, A:P197, A:G198, A:P199, A:G200, A:T201, A:Y202, A:G203, A:V204, A:D205, A:V206, A:V207, A:G208, A:Y209, A:D210, A:R211	55	0.692
4	A:V1, A:P2, A:S3, A:L4, A:P5, A:L6, A:V7, A:P8, A:L9, A:G10, A:E11, A:A12, A:A13, A:A14, A:K15, A:G16, A:I17, A:T18, A:A277, A:Y278, A:S280, A:I281, A:A284, A:A285, A:Y287, A:A288, A:A289, A:Y290, A:S291, A:V292, A:E293, A:D295, A:S304, A:G305, A:T306, A:H307, A:S308, A:W309, A:E310, A:Y311, A:A312, A:A313, A:Y314, A:S315, A:S316, A:L318, A:T319, A:A321, A:I322, A:A325, A:Y326, A:T328, A:D329, A:I330, A:N331, A:A332, A:F333, A:S334, A:V335, A:E336, A:A337, A:A338, A:A339, A:K340, A:P342	65	0.69

### Molecular docking

The protein–protein docking of the vaccine was carried out by numerous online tools for improving the accurateness of the prediction: i.e., ClusPro 2.0, PatchDock and HawkDock server. The docked complexes that were created by ClusPro 2.0 and PatchDock tools were further investigated by PRODIGY tool of HADDOCK web-server and FireDock server, respectively (Fig. 5B). The PRODIGY tool estimated the binding affinity score (kcal/mol) whereas FireDock predicted the global energy of the docked complexes. However, HawkDock produces ranking scores along with the binding free energy (kcal/mol). The binding free energy was deliberate after the MM-GBSA score in the HawkDock web-server. The vaccine in the docking experiment carried out by the ClusPro 2.0 and PRODIGY servers had the lowest binding affinity when docked with TLR-3 (− 32.82 kcal/mol). The vaccine with TLR-3 complex has good global binding energy score (− 35.88 kcal/mol) acquired from PatchDock server. However, the vaccine also indicated the best presentations with the nominated TLR-3 by the HawkDock server and also when studied in the MM-GBSA study with a relative binding free energy − 42.82 (kcal/mol) (Fig. 5C).

### Molecular dynamics simulation

**Figure 6:**
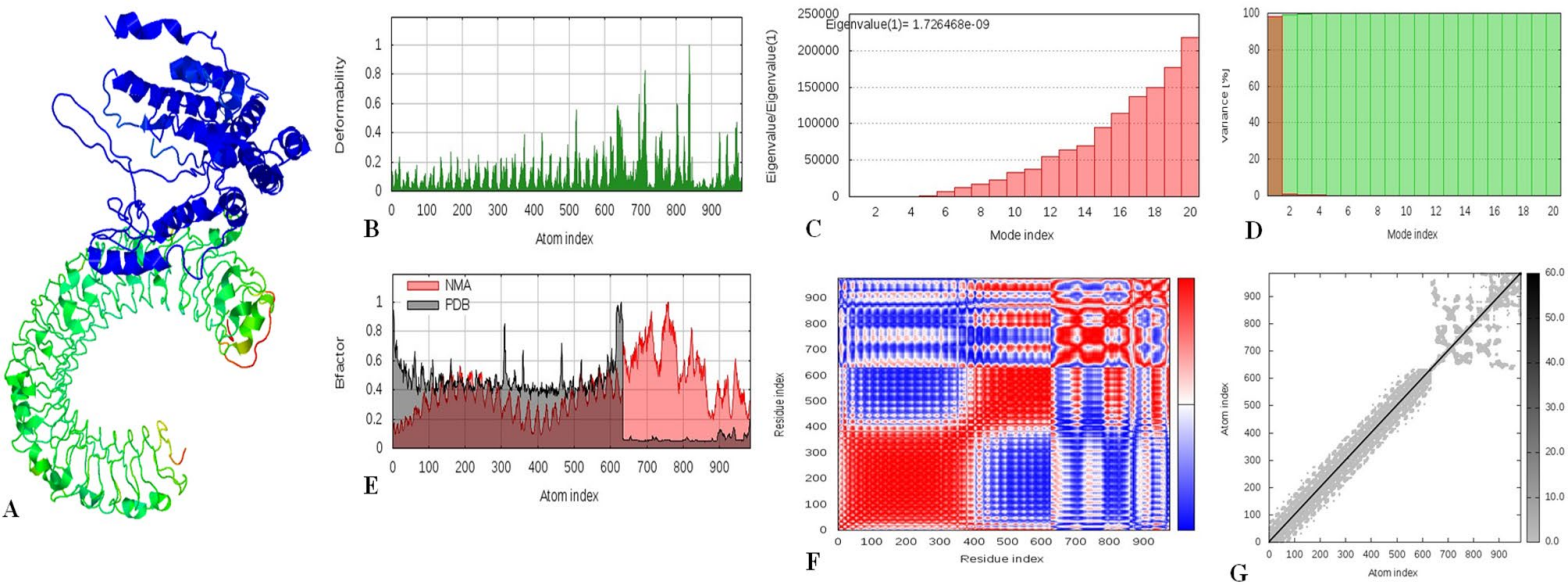
The results of molecular dynamics simulation of vaccine construct and TLR-3 docked complex. (**A**) NMA mobility, (**B**) deformability, (**C**) eigenvalues, (**D**) variance (red color indicates individual variances and green color indicates cumulative variances), (**E**) Bfactor, (**F**) co-variance map (correlated (red), uncorrelated (white) or anti-correlated (blue) motions) and (**G**) elastic network (darker gray regions indicate stiffer regions).

The results of molecular dynamics simulation and normal mode analysis (NMA) of vaccine construct and TLR-3 docked complex is illustrated in (Fig. 6A). The simulation study was conducted to determine the movements of molecules and atoms in the vaccine construct. The deformability graph of the complex illustrates the peaks in the graphs which represent the regions of the protein with deformability (Fig. 6B). The eigenvalue of the complex is 1.726468e−09 as shown in (Fig. 6C). The variance graph displays the cumulative variance by green colored and individual variance by red colored (Fig. 6D). The B-factor graph gives a clear visualization of the relation of the docked complex between the NMA and the PDB sector (Fig. 6E). The co-variance map of the complex where the correlated motion between a pair of residues is indicated by red color, uncorrelated by white color and anti-correlated by blue color (Fig. 6F). The complex 's elastic map shows the relation between the atoms and darker gray regions, indicating stiffer regions (Fig. 6G).

### Codon optimization and in-silico cloning

**Figure 7:**
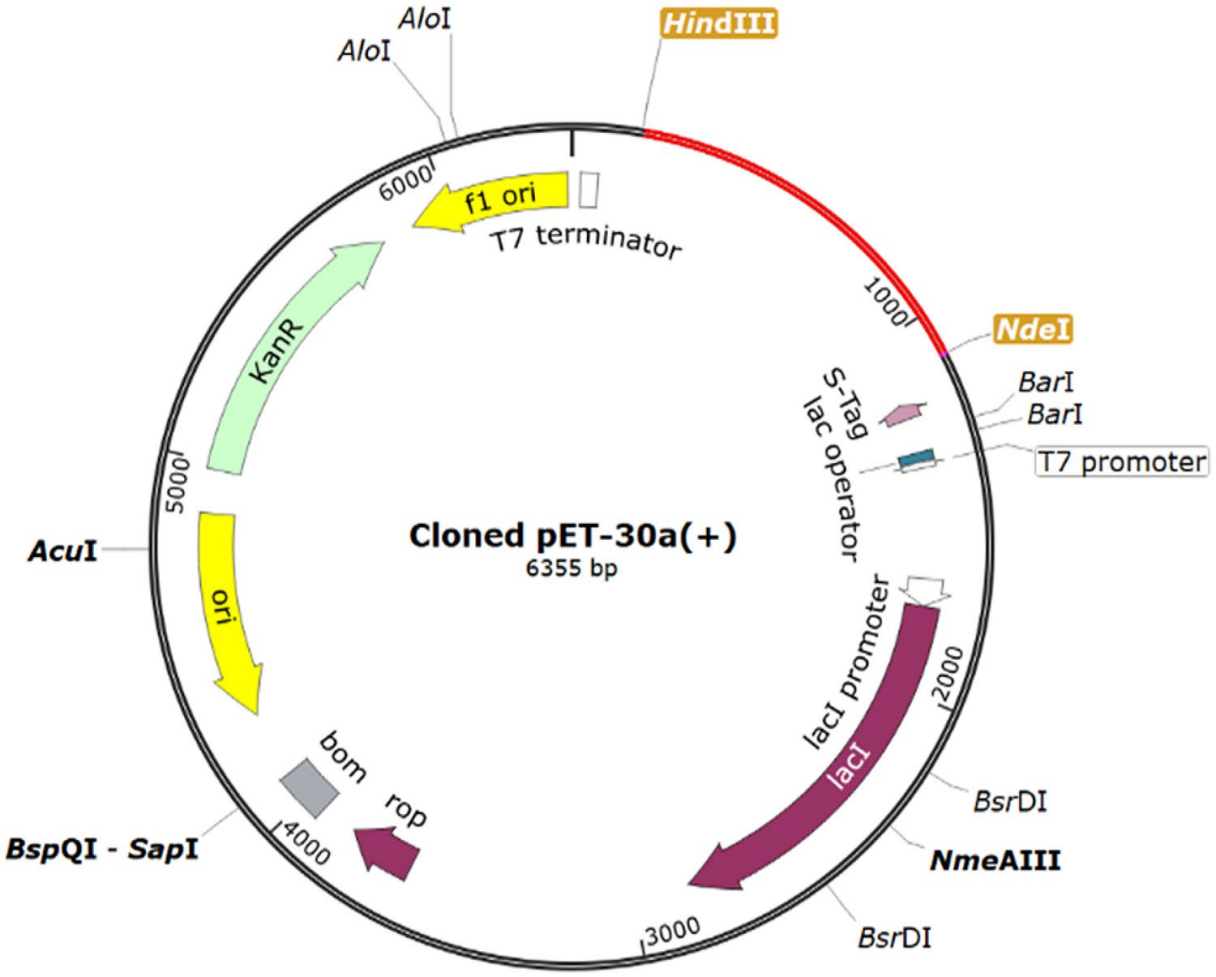
Expression vector pET30a (+). In silico restriction cloning of the multi-epitope vaccine sequence into the pET30a (+) expression vector using SnapGene software free-trial (https://www.snapgene.com/free-trial/), the red part represents the vaccine’s gene coding, and the black circle represents the vector backbone.

For optimizing the vaccine construct's codon usage, Java Codon Adaptation Tool (JCat) was used for maximal protein expression in *E. coli* (strain K12). The optimized codon sequence had a length of 927 nucleotides. The codon optimization index (CAI) value was predicted 1.0, and the average GC content of the adapted sequence was 59.2%, which indicates high expression in the *E. coli* host. Finally, the recombinant plasmid sequence was constructed by introducing the adapted codon sequences into the plasmid vector pET30a (+) using SnapGene software (Fig. 7).

### Immune simulation

**Figure 8:**
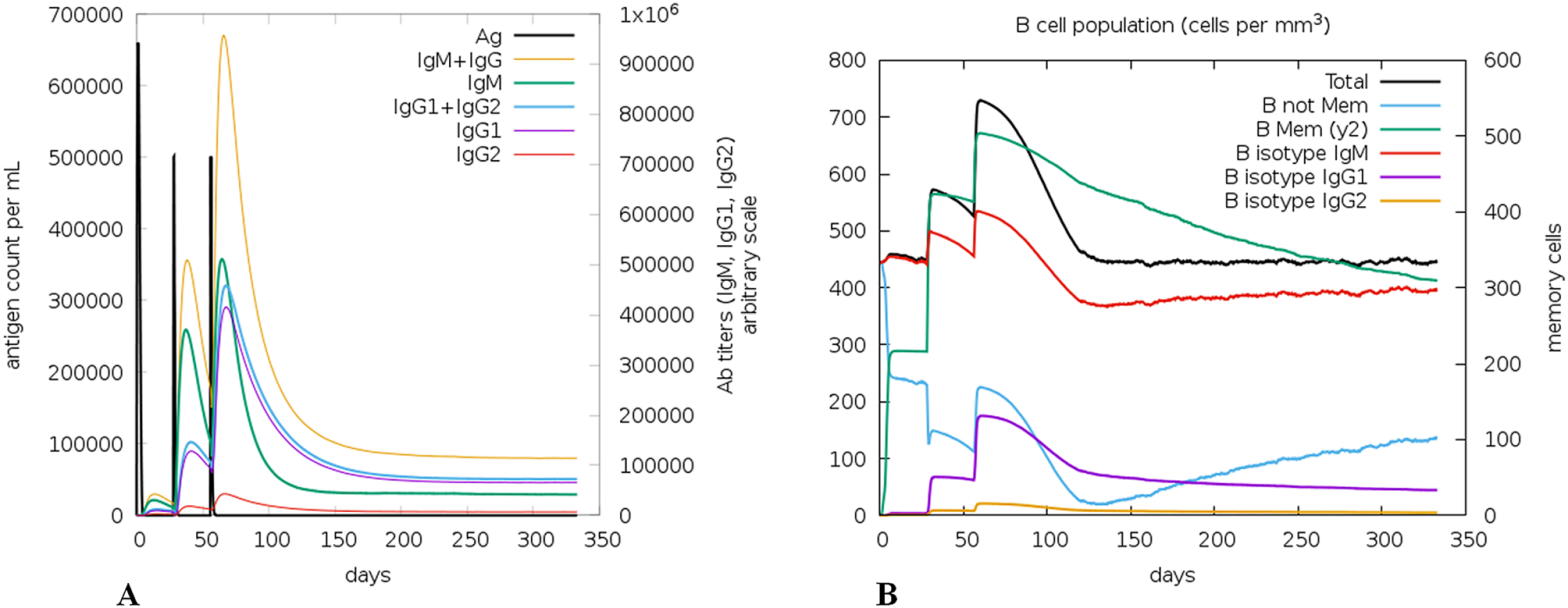
C-ImmSim presentation of an in silico immune simulation with the construct. (**A**) Immunoglobulin production in response to antigen injections (black vertical lines); specific subclasses are showed as colored peaks. (**B**) The evolution of B-cell populations after the three injections.

**Figure 9:**
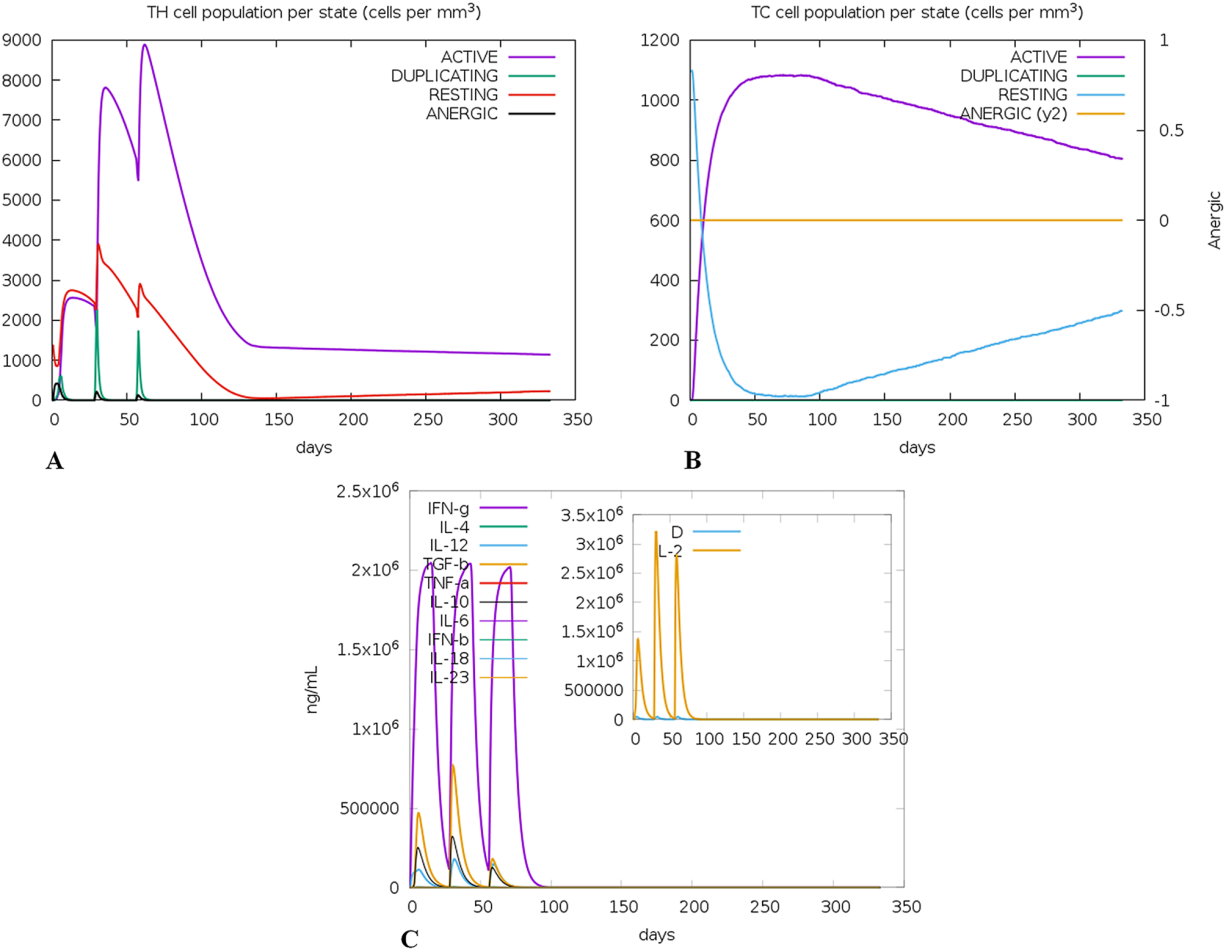
C-ImmSim presentation of an in silico immune simulation with the construct. (**A**) The evolution of T-helper, and (**B**) T-cytotoxic cell populations per state after the injections. The resting state represents cells not presented with the antigen while the anergic state characterizes tolerance of the T-cells to the antigen due to repeated exposures. (**C**) The main plot shows cytokine levels after the injections. The insert plot shows IL-2 level with the Simpson index, D shown by the dotted line. D is a measure of diversity. Increase in D over time indicates emergence of different epitope-specific dominant clones of T-cells. The smaller the D value, the lower the diversity.

C-ImmSim studies the successive and effective immune responses of the state of the cell and the memory of immune cells by a mechanism that increases their half-life. The effect of the approach is that few cells substantially increase their half-life and live longer than other cells. ImmSim server immune simulation outcomes confirmed consistency with real immune reactions. The primary response was illustrated by high IgM levels. In addition, an increase in the B-cell population was characterized as an increase in immunoglobulin expression (IgG1+IgG2, IgM, and IgG+IgM), resulting in a decrease in antigen concentration (Fig. 8A,B). There is also a clear increase in the population of Th (helper) and T C (cytotoxic) cells with memory growth (Fig. 9A,B). IFN-γ production was also identified to have been stimulated after immunization (Fig. 9C). The T cell population results were significantly approachable as the memory developed, and all other immune cell populations were exposed to be consistent.

## Discussion

Tuberculosis (TB) is a life threating disease and the TB vaccines used globally, the BCG vaccine, offers limited protection against TB for children, and adults, which accounts for most of the TB cases worldwide^66^. Therefore, new candidate vaccine against TB are extremely needed and some of them are under evaluation in clinical trials. Development in reverse vaccinology and the existence of genomics and proteomics information help in vaccine designing. Moreover, successful implementation of the bio-informatics tools is beneficial compared to traditional vaccine design^67^. Identification of immunogenic antigens is an essential step in vaccine designing as it can be potentially used for in-silico epitope prediction^68^. Immunogenic antigens have a unique attribute to attach and respond with immune cells and perform as immuno-dominant^69^. Using computational methods, we conducted an organized and complete valuation of the four Mtb antigens (Rv2608, Rv2684, Rv3804c, and Rv0125) constituting Mtb subunit vaccines undergoing clinical trials. We predicted linear B and T-cell epitopes using bioinformatics tools that could possibly stimulate both cellular and humoral immunity^32^. The prediction of linear B and T-cell epitopes from Mtb was selected to predict the vaccines as they play an important role in the cellular and biological developments. These epitopes of B-cells and T-cells may theoretically be used to produce vaccines targeting Mtb and may be reliable for stimulating both humoral and cell-mediated immunity. Prediction of B-cell epitopes is a significant characteristic for the designing of vaccines^70^, and to provide the site for the antigen–antibody interactions^71–73^. In the present research, 16-mer B-cell epitopes were predicted via the ABCpred server.

T-cell epitopes are essential for adaptive immune stimulation and are sufficient to cooperate with MHC molecules^74^. Therefore, the selection of epitopes fixes with MHC is an essential aspect in predicting potent T-cell epitopes^75^. Also, CD4^+^ and CD8^+^ T-cells' recognition is critical while developing multi-epitope-based vaccines^76,77^. We predicted B and T-cell epitopes from the nominated antigens and joined them using AAY and GPGPG linkers in order to construct epitope-based vaccine^78^. To generate sequences with reduced junctional immunogenicity, the previously described GPGPG and AAY linkers^57,63^ were integrated between the selected epitopes, enabling the rational design of a multi-epitope vaccine^78^. The EAAAK linker^79^ was also fused between the adjuvant and the epitopes sequences for a best expression and bioactivity improvement of vaccine. Immuno-informatics evaluation of the constructed vaccine specified many MHC Class I, MHC Class II, IFN-γ, and linear B-cell epitopes. However, previous studies recommends that IFN-γ also promotes general protection against TB in the mice lungs^80^. The lack of the protein vaccine's allergenic function has nearer enhanced its efficacy as a candidate vaccine. The constructed multi-epitope vaccine showed higher scores of antigenicity both on ANTIGENpro and Vaxijen v2.0 server. Multi-epitopic vaccines have less immunogenicity and need adjuvants^78^.

The final protein’s molecular weight (MW) was predicted to be 31.9 kDa and has been estimated to be highly soluble upon expression, along with its virtual immunogenicity. The solubility of recombinant protein overexpressed in the *E. coli* host is critical for numerous biochemical and functional studies^57^. The estimated theoretical pI is 4.28, suggesting that the constructed vaccine is acidic. The predicted score of the instability index was 25.51, which suggests that the protein would be extremely stable upon expression, thus further firming its probability. The aliphatic index shows that there are aliphatic side chains in the protein, representing possible hydrophobicity. Knowledge about the target protein's secondary and tertiary structures is vital in vaccine development^78^. Analyses of the secondary structure showed that the protein contained mainly of 58% coils, with 13% of residues disordered. Natively unfolded protein regions and α-helical coiled-coils peptides have been identified as significant “structural antigens” types. When examined in synthetic peptides, these two structural types can fold into their native structure and thus be recognized by antibodies naturally induced in response to infection^81^. The vaccine candidate's 3D structure improved considerably after the refinement and displayed appropriate characteristics based on the Ramachandran plot's results. The result of the Ramachandran plot indicates that 85.9% of the residues are initiate in the favored regions, and 8.9% are allowed regions with less (5.2%) residues in the outlier region; this suggests that the quality of the whole model is acceptable. One of the primary feature in validating a candidate vaccine is to screen for immunoreactivity over serological study^82^.

Further, to examine the capability of the constructed vaccine to bind with TLR on immune cells, the TLR-3 was docked with the vaccine. The results revealed that the constructed vaccine had a high binding affinity towards TLR-3. This interface of vaccine with TLR-3 was signifying that vaccine have the probability to produce both innate and adaptive immune response. For exploring the stability and dynamics performance of the TLR-3-vaccine docked complex, MD simulation was implemented, and the RMSD plot representing the steady binding of the complex.

Immune simulation showed results consistent with typical immune responses. Following repeated exposure to the antigen, there was a general increase in the generated immune responses. The development of memory B-cells and T- cells was evident, with memory in B-cells lasting several months. Helper T cells were particularly stimulated. Another interesting observation was that levels of IFN-γ and IL-2 rose after the first injection and remained at peak levels following repeated exposures to the antigen. This indicates high levels of T H cells and consequently efficient Ig production, supporting a humoral response. The Simpson index, D for investigation of clonal specificity suggests a possible diverse immune response.

This needs that recombinant protein be expressed in a suitable host. The preferred alternative for the expression of recombinant proteins is the *E. coli* expression systems^83,84^. Codon optimization was carried out to get high-level expression of the recombinant vaccine in *E. coli* system (K12 strain). For high-level protein expression in bacteria, both the GC content (59.2%) and the CAI score (1.0) were favorable. The next step currently being planned is to express this peptide in a bacterial system and carry out the numerous immunological analyses required to confirm the results achieved through immuno-informatics analysis.

## Conclusion

Computational approaches presented in the present work may produce new knowledge about Mtb vaccine antigens and new vaccine candidates that cannot simply be acquired from pre-clinical, in-vitro and animal studies. This study used an immuno-informatics tool to define tuberculosis novel multi-epitope subunit vaccine, which is highly immunogenic and has appropriate properties to be a carrier vaccine. Epitope-prediction tools were used to analyze multiple B-cell and T-cell epitopes, which were fused using suitable linkers and adjuvant to enhance the vaccine’s immunogenicity. Antigenicity, allergenicity, solubility, as well as physiochemical properties and tertiary structure analysis, of vaccine were found to be very satisfactory. Molecular docking and molecular dynamics simulation analysis of TLR-3 and vaccine were completed, allowing estimation of the binding affinity and stability of the complex. The in silico immune simulation confirmed immune cell response against antigen clearance rate. The codon optimization also provided an optimistic CAI value, which will help in-vivo expression studies soon. In this research, we made use of various immune-informatics tools for investigating different properties of vaccine. Additionally, the predicted epitopes-based subunit vaccine's assessment is hugely acceptable to prove them as an immunogenic and potential vaccine candidate against tuberculosis.

## References

[1] Minch KJ (2015). The DNA-binding network of *Mycobacterium tuberculosis*. Nat. Commun..

[2] Maji A (2018). Gut microbiome contributes to impairment of immunity in pulmonary tuberculosis patients by alteration of butyrate and propionate producers. Environ. Microbiol..

[3] Khan PY (2019). Transmission of drug-resistant tuberculosis in HIV-endemic settings. Lancet. Infect. Dis.

[4] Singhvi N (2020). Interplay of human gut microbiome in health and wellness. Indian J. Microbiol..

[5] Prabowo SA (2019). Historical BCG vaccination combined with drug treatment enhances inhibition of mycobacterial growth ex vivo in human peripheral blood cells. Sci. Rep..

[6] Dalsass M, Brozzi A, Medini D, Rappuoli R (2019). Comparison of open-source reverse vaccinology programs for bacterial vaccine antigen discovery. Front. Immunol..

[7] Méndez-Samperio P (2016). Global efforts in the development of vaccines for tuberculosis: Requirements for improved vaccines against *Mycobacterium tuberculosis*. Scand. J. Immunol..

[8] Méndez-Samperio, P. (Future Medicine, 2016).

[9] Zenteno-Cuevas R (2017). Successes and failures in human tuberculosis vaccine development. Expert Opin. Biol. Ther..

[10] Bali P, Tousif S, Das G, Van Kaer L (2015). Strategies to improve BCG vaccine efficacy. Immunotherapy.

[11] Romano M, Huygen K (2012). An update on vaccines for tuberculosis—there is more to it than just waning of BCG efficacy with time. Expert Opin. Biol. Ther..

[12] Detmer A, Glenting J (2006). Live bacterial vaccines—a review and identification of potential hazards. Microb. Cell Fact..

[13] Evans TG, Schrager L, Thole J (2016). Status of vaccine research and development of vaccines for tuberculosis. Vaccine.

[14] Wilkie MEM, McShane H (2015). TB vaccine development: Where are we and why is it so difficult?. Thorax.

[15] Gillard P (2016). Safety and immunogenicity of the M72/AS01E candidate tuberculosis vaccine in adults with tuberculosis: A phase II randomised study. Tuberculosis.

[16] Kagina BMN (2014). The novel tuberculosis vaccine, AERAS-402, is safe in healthy infants previously vaccinated with BCG, and induces dose-dependent CD4 and CD8T cell responses. Vaccine.

[17] Li W, Joshi MD, Singhania S, Ramsey KH, Murthy AK (2014). Peptide vaccine: Progress and challenges. Vaccines.

[18] Slingluff CL (2011). The present and future of peptide vaccines for cancer: Single or multiple, long or short, alone or in combination?. Cancer J. (Sudbury Mass.).

[19] Ong E, He Y, Yang Z (2020). Epitope promiscuity and population coverage of *Mycobacterium tuberculosis* protein antigens in current subunit vaccines under development. Infect. Genet. Evol..

[20] Lindestam Arlehamn CS (2016). A quantitative analysis of complexity of human pathogen-specific CD4 T cell responses in healthy *M. tuberculosis* infected South Africans. PLoS Pathog..

[21] Rodo MJ (2019). A comparison of antigen-specific T cell responses induced by six novel tuberculosis vaccine candidates. PLoS Pathog..

[22] Luabeya AKK (2015). First-in-human trial of the post-exposure tuberculosis vaccine H56: IC31 in *Mycobacterium tuberculosis* infected and non-infected healthy adults. Vaccine.

[23] Penn-Nicholson A (2018). Safety and immunogenicity of the novel tuberculosis vaccine ID93+ GLA-SE in BCG-vaccinated healthy adults in South Africa: A randomised, double-blind, placebo-controlled phase 1 trial. Lancet Respir. Med..

[24] Suliman S (2019). Dose optimization of H56: IC31 vaccine for tuberculosis-endemic populations. A double-blind, placebo-controlled, dose-selection trial. Am. J. Respir. Crit. Care Med..

[25] Kumar, V. & Jena, M. Reverse vaccinology approach towards the in-silico multiepitope vaccine development against SARS-CoV-2. (2020). 10.12688/f1000research.36371.1 PMC8009247 33841800

[26] Rahman MS (2020). Epitope-based chimeric peptide vaccine design against S, M and E proteins of SARS-CoV-2 etiologic agent of global pandemic COVID-19: An in silico approach. PeerJ.

[27] Rahman N (2020). Vaccine design from the ensemble of surface glycoprotein epitopes of SARS-CoV-2: An immunoinformatics approach. Vaccines.

[28] Ullah A, Sarkar B, Islam SS (2020). Exploiting the reverse vaccinology approach to design novel subunit vaccine against ebola virus. Immunobiology.

[29] Damfo SA, Reche P, Gatherer D, Flower DR (2017). In silico design of knowledge-based Plasmodium falciparum epitope ensemble vaccines. J. Mol. Graph. Model..

[30] Solanki V, Tiwari V (2018). Subtractive proteomics to identify novel drug targets and reverse vaccinology for the development of chimeric vaccine against *Acinetobacter baumannii*. Sci. Rep..

[31] Ahmadi K (2019). Epitope-based immunoinformatics study of a novel Hla-MntC-SACOL0723 fusion protein from *Staphylococcus aureus*: Induction of multi-pattern immune responses. Mol. Immunol..

[32] Yazdani Z (2020). Designing a potent L1 protein-based HPV peptide vaccine: A bioinformatics approach. Comput. Biol. Chem..

[33] Watt J, Liu J (2020). Preclinical progress of subunit and live attenuated *Mycobacterium tuberculosis* vaccines: A review following the first in human efficacy trial. Pharmaceutics.

[34] Skwark MJ (2019). Mabellini: A genome-wide database for understanding the structural proteome and evaluating prospective antimicrobial targets of the emerging pathogen *Mycobacterium abscessus*. Database.

[35] Kapopoulou A, Lew JM, Cole ST (2011). The MycoBrowser portal: A comprehensive and manually annotated resource for mycobacterial genomes. Tuberculosis.

[36] Larsen MV (2007). Large-scale validation of methods for cytotoxic T-lymphocyte epitope prediction. BMC Bioinform..

[37] Peters B, Bulik S, Tampe R, Van Endert PM, Holzhütter H-G (2003). Identifying MHC class I epitopes by predicting the TAP transport efficiency of epitope precursors. J. Immunol..

[38] Zhang Q (2008). Immune epitope database analysis resource (IEDB-AR). Nucleic Acids Res..

[39] Dhanda SK, Vir P, Raghava GPS (2013). Designing of interferon-gamma inducing MHC class-II binders. Biol. Direct.

[40] Saha S, Raghava GPS (2006). Prediction of continuous B-cell epitopes in an antigen using recurrent neural network. Prot. Struct. Funct. Bioinform..

[41] Magnan CN (2010). High-throughput prediction of protein antigenicity using protein microarray data. Bioinformatics.

[42] Doytchinova IA, Flower DR (2007). VaxiJen: A server for prediction of protective antigens, tumour antigens and subunit vaccines. BMC Bioinform..

[43] Pandey RK, Ojha R, Aathmanathan VS, Krishnan M, Prajapati VK (2018). Immunoinformatics approaches to design a novel multi-epitope subunit vaccine against HIV infection. Vaccine.

[44] Dimitrov I, Bangov I, Flower DR, Doytchinova I (2014). AllerTOP v.2—a server for in silico prediction of allergens. J. Mol. Model..

[45] Dimitrov I, Naneva L, Doytchinova I, Bangov I (2014). AllergenFP: Allergenicity prediction by descriptor fingerprints. Bioinformatics.

[46] Shahid F, Ashfaq UA, Javaid A, Khalid H (2020). Immunoinformatics guided rational design of a next generation multi epitope based peptide (MEBP) vaccine by exploring Zika virus proteome. Infect. Genet. Evol..

[47] Gupta S (2013). In silico approach for predicting toxicity of peptides and proteins. PLoS One.

[48] Kling A (2015). Targeting DnaN for tuberculosis therapy using novel griselimycins. Science.

[49] Gasteiger E (2005). The Proteomics Protocols Handbook.

[50] Hebditch M, Carballo-Amador MA, Charonis S, Curtis R, Warwicker J (2017). Protein–Sol: A web tool for predicting protein solubility from sequence. Bioinformatics.

[51] McGuffin LJ, Bryson K, Jones DT (2000). The PSIPRED protein structure prediction server. Bioinformatics.

[52] Wang S, Peng J, Ma J, Xu J (2016). Protein secondary structure prediction using deep convolutional neural fields. Sci. Rep..

[53] Yang J (2015). The I-TASSER Suite: Protein structure and function prediction. Nat. Methods.

[54] Zhang Y, Skolnick J (2007). Scoring function for automated assessment of protein structure template quality. Prot. N. Y..

[55] Shey RA (2019). In-silico design of a multi-epitope vaccine candidate against onchocerciasis and related filarial diseases. Sci. Rep..

[56] Heo L, Park H, Seok C (2013). GalaxyRefine: Protein structure refinement driven by side-chain repacking. Nucleic Acids Res..

[57] Khatoon N, Pandey RK, Prajapati VK (2017). Exploring Leishmania secretory proteins to design B and T cell multi-epitope subunit vaccine using immunoinformatics approach. Sci. Rep..

[58] Wiederstein M, Sippl MJ (2007). ProSA-web: Interactive web service for the recognition of errors in three-dimensional structures of proteins. Nucleic Acids Res..

[59] Lovell SC (2003). Structure validation by Cα geometry: ϕ, ψ and Cβ deviation. Prot. Struct. Funct. Bioinform..

[60] Bhati S, Kaushik V, Singh J (2019). In silico identification of piperazine linked thiohydantoin derivatives as novel androgen antagonist in prostate cancer treatment. Int. J. Pept. Res. Ther..

[61] Weng G (2019). HawkDock: A web server to predict and analyze the protein–protein complex based on computational docking and MM/GBSA. Nucleic Acids Res..

[62] Lopéz-Blanco JR, Garzón JI, Chacón P (2011). iMod: Multipurpose normal mode analysis in internal coordinates. Bioinformatics.

[63] Ali M (2017). Exploring dengue genome to construct a multi-epitope based subunit vaccine by utilizing immunoinformatics approach to battle against dengue infection. Sci. Rep..

[64] Peele KA, Srihansa T, Krupanidhi S, Sai AV, Venkateswarulu T (2020). Design of multi-epitope vaccine candidate against SARS-CoV-2: A in-silico study. J. Biomol. Struct. Dyn..

[65] Ikai A (1980). Thermostability and aliphatic index of globular proteins. J. Biochem..

[66] Riccomi A (2019). Parenteral vaccination with a tuberculosis subunit vaccine in presence of retinoic acid provides early but transient protection to *M. tuberculosis* infection. Front. Immunol..

[67] Reginald K, Chan Y, Plebanski M, Poh CL (2018). Development of peptide vaccines in dengue. Curr. Pharm. Des..

[68] Bahrami AA, Payandeh Z, Khalili S, Zakeri A, Bandehpour M (2019). Immunoinformatics: In silico approaches and computational design of a multi-epitope, immunogenic protein. Int. Rev. Immunol..

[69] Galluzzi L, Buqué A, Kepp O, Zitvogel L, Kroemer G (2017). Immunogenic cell death in cancer and infectious disease. Nat. Rev. Immunol..

[70] Dubey KK (2018). Vaccine and antibody production in plants: Developments and computational tools. Brief. Funct. Genom..

[71] Lu LL, Suscovich TJ, Fortune SM, Alter G (2018). Beyond binding: Antibody effector functions in infectious diseases. Nat. Rev. Immunol..

[72] El-Manzalawy Y, Dobbs D, Honavar V (2008). Predicting linear B-cell epitopes using string kernels. J. Mol. Recogn. Interdiscip. J..

[73] Krocova Z (2020). The role of B cells in an early immune response to *Mycobacterium bovis*. Microb. Pathog..

[74] Verma S, Singhvi N, Singh Y, Shukla P (2020). Computational approaches in epitope design using DNA binding proteins as vaccine candidate in *Mycobacterium tuberculosis*. Infect. Genet. Evol..

[75] Eickhoff CS (2019). Highly conserved influenza T cell epitopes induce broadly protective immunity. Vaccine.

[76] Patankar YR (2019). Limited recognition of *Mycobacterium tuberculosis*-infected macrophages by polyclonal CD4 and CD8 T cells from the lungs of infected mice. Mucosal Immunol..

[77] Russell SL (2019). Compromised metabolic reprogramming is an early indicator of CD8+ T cell dysfunction during chronic *Mycobacterium tuberculosis* infection. Cell Rep..

[78] Meza B, Ascencio F, Sierra-Beltrán AP, Torres J, Angulo C (2017). A novel design of a multi-antigenic, multistage and multi-epitope vaccine against *Helicobacter pylori*: An in silico approach. Infect. Genet. Evol..

[79] Arai R, Ueda H, Kitayama A, Kamiya N, Nagamune T (2001). Design of the linkers which effectively separate domains of a bifunctional fusion protein. Protein Eng..

[80] Sakai S (2016). CD4 T cell-derived IFN-γ plays a minimal role in control of pulmonary *Mycobacterium tuberculosis* infection and must be actively repressed by PD-1 to prevent lethal disease. PLoS Pathog..

[81] Corradin G, Villard V, Kajava AV (2007). Protein structure based strategies for antigen discovery and vaccine development against malaria and other pathogens. Endocrine Metab. Immune Disord. Drug Targets (Formerly Curr. Drug Targets Immune Endocrine Metab, Disord.).

[82] Gori A, Longhi R, Peri C, Colombo G (2013). Peptides for immunological purposes: Design, strategies and applications. Amino Acids.

[83] Chen R (2012). Bacterial expression systems for recombinant protein production: *E. coli* and beyond. Biotechnol. Adv..

[84] Rosano GL, Ceccarelli EA (2014). Recombinant protein expression in *Escherichia coli*: Advances and challenges. Front. Microbiol..

